# Non-equilibrium diffusive fluxes of ions and their impact on the refractory period of tightly packed neurons

**DOI:** 10.1186/1471-2202-12-S1-P243

**Published:** 2011-07-18

**Authors:** Jack Wilson, Sorinel A Oprisan

**Affiliations:** 1 Department of Psychology, College of Charleston, Charleston, SC 29424, USA; 2 Department of Physics and Astronomy, College of Charleston, Charleston, SC 29424, USA

## 

Action potentials (APs) generated and sustained by ionic currents flowing through neural cell’s membrane constitute the main way these cells communicate with each other. The most common, although not the only possible mechanism of generating APs, involves sodium influx that ensures cell depolarization and potassium efflux that ensures cell repolaroization. A sodium-potassium exchanger and leakage currents eliminate the ionic disturbance created by an AP. In a closely packed bundle of neuronal cells with fast diffusion of ionic species in the extracellular space (ECS) an AP generated in one axon could impact the excitability of nearby neurons. The ECS is similar in composition to the cerebrospinal fluid that baths the outer surface of the brain and is present in large internal cavities or ventricles and in the spinal canal. The ionic composition of ECS is mostly made of sodium and chloride dissolved in water with much smaller amounts of other substances, including potassium and calcium. Previous studies have considered the role of potassium in seizures by employing a computational model of potassium diffusion between periodically firing neurons [1]. However, absent from most of these studies on diffusion coupling is the potential effect of sodium dynamics on this form of neuronal coupling [2]. Based on previously implemented models that include detailed balances of the main ionic species, we modeled a Hodgkin-Huxley cell able to generate periodic spiking or bursting APs. The model accounts for the dynamics of major (sodium and potassium) ionic species, as well as certain aspects of the extracellular environment (diffusion coefficients, the intracellular/extracellular pace ratio, etc.) The results from the full model were compared to reduced models, in which the dynamics of only one ionic species is considered, through analysis of refractory period induced by a simulated injected current into the neuron. Our results suggest that the refractory period and, therefore, the ability of a neuron to produce regenerative APs depends strongly on the firing rate of nearby neurons and the parameters of the extracellular space (diffusion coefficients and extracellular/intracellular space ratio).
